# Can Bioactive Food Substances Contribute to Cystic Fibrosis-Related Cardiovascular Disease Prevention?

**DOI:** 10.3390/nu15020314

**Published:** 2023-01-08

**Authors:** Laura Mihaela Trandafir, Otilia Elena Frăsinariu, Elena Țarcă, Lăcrămioara Ionela Butnariu, Maria Magdalena Leon Constantin, Mihaela Moscalu, Oana Raluca Temneanu, Alina Sinziana Melinte Popescu, Marian George Melinte Popescu, Iuliana Magdalena Stârcea, Elena Cojocaru, Stefana Maria Moisa

**Affiliations:** 1 Department of Mother and Child Medicine–Pediatrics, “Grigore T. Popa” University of Medicine and Pharmacy, 700115 Iaşi, Romania; 2 Department of Surgery II-Pediatric Surgery, “Grigore T. Popa” University of Medicine and Pharmacy, 700115 Iaşi, Romania; 3 Department of Medical Genetics, Faculty of Medicine, “Grigore T. Popa” University of Medicine and Pharmacy, 700115 Iași, Romania; 4 Medical I Department, “Grigore T. Popa” University of Medicine and Pharmacy, 700115 Iaşi, Romania; 5 Department of Preventive Medicine and Interdisciplinarity, “Grigore T. Popa” University of Medicine and Pharmacy, 700115 Iași, Romania; 6 Department of General Nursing, Faculty of Medicine and Biological Sciences, “Ştefan cel Mare” University of Suceava, 720229 Suceava, Romania; 7 Department of Morphofunctional Sciences I–Pathology, “Grigore T. Popa” University of Medicine and Pharmacy, 700115 Iaşi, Romania

**Keywords:** cystic fibrosis, cardiovascular risk factors, nutrition, bioactive food components

## Abstract

Advances in cystic fibrosis (CF) care have significantly improved the quality of life and life expectancy of patients. Nutritional therapy based on a high-calorie, high-fat diet, antibiotics, as well as new therapies focused on CFTR modulators change the natural course of the disease. They do so by improving pulmonary function and growing BMI. However, the increased weight of such patients can lead to unwanted long-term cardiovascular effects. People with CF (pwCF) experience several cardiovascular risk factors. Such factors include a high-fat diet and increased dietary intake, altered lipid metabolism, a decrease in the level of fat-soluble antioxidants, heightened systemic inflammation, therapeutic interventions, and diabetes mellitus. PwCF must pay special attention to food and eating habits in order to maintain a nutritional status that is as close as possible to the proper physiological one. They also have to benefit from appropriate nutritional counseling, which is essential in the evolution and prognosis of the disease. Growing evidence collected in the last years shows that many bioactive food components, such as phytochemicals, polyunsaturated fatty acids, and antioxidants have favorable effects in the management of CF. An important positive effect is cardiovascular prevention. The possibility of preventing/reducing cardiovascular risk in CF patients enhances both quality of life and life expectancy in the long run.

## 1. Introduction

Cystic fibrosis (CF) is the most common severe autosomal recessive genetic disease in Caucasians caused by a mutation of the cystic fibrosis transmembrane conductance regulator (*CFTR*) gene. CF is classically considered a multi-system disease, with such symptoms including chronic inflammation, recurrent respiratory infections, gastrointestinal disease, and premature mortality. Advances in CF care have significantly improved the quality of life and life expectancy of patients. Diagnosing CF by neonatal screening allows for the early use of nutritional therapy based on high-calorie supplementation with no restriction on fat intake and pancreatic enzyme substitutive treatment to prevent malnutrition. Currently, CF-related malnutrition due to both intestinal malabsorption and increased energy expenditure has significantly decreased because of targeted interventions [1,2,3]. Antibiotics and physiotherapy use have limited pulmonary infections and overall pulmonary dysfunction. Moreover, new therapies targeting the CFTR regulator protein change the natural course of the disease by improving pulmonary function [1]. These factors have led to an increase in affected individuals’ life expectancy. According to data reported from the registries of the US CF Foundation and the European CF Society, median survival age for pwCF varies from 44 to 53 years [4].

On the other hand, in consideration of the increase in life expectancy, overweight and obesity are progressively becoming a source of concern for pwCF [5]. Based on data from the 2021 CF Patient Registry Annual Data Report, 40.4 percent of adults were overweight or obese, as compared to 15.3 percent in 2001 [6]. In the general population, overweight and obesity are considered relevant risk factors for cardiovascular complications including hypertension and ischemic heart disease. Over the past several years, overweight and obesity have been recognized as potential clinical issues for pwCF. Therefore, the risk of obesity-associated complications, including cardiovascular disease (CVD), is increasing in these individuals [3].

*The aim of our study* is to highlight, through a review of the literature, those nutritional risk factors involved in the occurrence of cardiomyopathy in patients with cystic fibrosis, to draw attention to the increasing risk of obesity in these patients, and to review the methods of treatment and prevention of this disease. We used the PubMed engine for our study and searched for cystic fibrosis articles (systematic reviews, randomized controlled trials, observational studies, series of cases studies and case reports), from the earliest time possible until October 2022; 59,822 results were returned. Then, we used the Medical Subject Headings or MeSH terms “cardiomyopathy”, “obesity”, and “nutrition” for selecting the appropriate articles for our review. In the mentioned order, 77, 270 and 2145 articles were found. We reviewed the titles and the available abstracts of the papers from the last 22 years (1774 articles) and the full text of 827 articles to extract data for our review. One hundred eighty-two relevant citations were included in our reference list.

## 2. Cardiovascular Disease in Patients with Cystic Fibrosis

CF is a complex multi-system chronic disease characterized by progressive organ dysfunction, possibly involving the heart [7]. In the past, CVD in pwCF used to be limited to right ventricular dysfunction because of end-stage chronic obstructive pulmonary disease. Existing data regarding cardiac manifestations are conflicting, and it is unclear whether cardiac abnormalities, if present, are related to disease severity [5,8]. As life expectancy is improving, understanding cardiac involvement in CF is important in terms of quality of life, prognosis, and transplant candidacy [9,10].

Recent epidemiological studies showed an increasing recognition of CF phenotypes that can increase cardiovascular risk, especially in adults. A high-calorie, high-fat diet and various therapies have led to obesity, systemic arterial hypertension, and chronic kidney disease in this age group [8,11,12].

The use of systemic and enteric antibiotics throughout the life modifies the intestinal microbiome and related metabolic functions [13,14]. The mechanisms by which antibiotic-induced dysbiosis in pwCF lead to specific cardiovascular alterations are still unclear.

Future studies will contribute to improving therapeutic interventions from childhood. Furthermore, patients receiving new CFTR therapy showed increasing body mass index, blood pressure and serum lipids, especially an increase in low-density lipoprotein [1,15].

The identification of CFTR on human myocytes has prompted investigation of a possible CF-related cardiomyopathy, possibly starting from childhood, which influences systolic and diastolic heart function [7,16]. Current CF patient care guidelines do not include recommendations regarding the early identification and prevention of CVD, as opposed to other complications such as hepatic disease. The 2019 ACC/AHA Guideline on the Primary Prevention of Cardiovascular Disease (made for the general adult population) states that with optimal primordial and primary prevention, CVD is “largely preventable,” and that earlier interventions are likely to be more effective [17]. This paradigm is particularly relevant to pwCF, but it has yet to be incorporated into clinical care for CF as it has in other chronic inflammatory conditions [17]. Cardiovascular damage in CF is related to both the heart and the vascular bed, causing a wide array of symptoms that need proper management (Table 1).

### 2.1. Cardiomyopathy

Chronic inflammation is thought to be a cornerstone for the myocardial changes that accompany CF, as chronic myocardial inflammation leads to myocardial fibrosis and its mechanical, electrical, and vasomotor consequences [21]. Several studies point towards a myocardial involvement, the so-called CF-related cardiomyopathy accompanied by systolic and diastolic dysfunction that seems to set in during childhood [22]. Chronic inflammation is not the only trigger for myocardial dysfunction, as chronic hypoxia, myocardial fibrosis, and cardiomyocyte contraction regulation linked to the CFTR also seem to play a role [23,24,25].

There are several physio-pathological explanations of myocardial dysfunction in CF patients. One is chronic hypoxia leading to myocardial fibrosis [26]. Another is the presence of pulmonary hypertension causing right ventricular overload, remodeling (hypertrophy and dilatation), and functional impairment. Proinflammatory molecules (TNF alpha, interleukins 1 and 6) impaired myocyte metabolism, causing muscular hypertrophy and disturbing inotropism [27]. Furthermore, the profibrotic factors aldosterone and angiotensin II plasma levels may be increased in CF patients [28]. Diabetic cardiomyopathy may also play a role in CF patients’ ventricular dysfunction by inducing myocardial stiffness, interstitial fibrosis, neuropathy, and small vessel disease. Finally, CFTR may play a role in regulating the action potential duration [25].

### 2.2. Right and Left Ventricular Dysfunction

Two hypotheses explain myocardial fibrosis and necrosis in pwCF, leading to ventricular dysfunction [29,30]. According to the first theory, myocardial cytolysis would result from activation of the kinin system by activated pancreatic enzymes (kallikrein and trypsin), which are released into the circulation from a diseased pancreas. The second theory claims that myocardial necrosis can be caused by the deficiency of vitamins E, B1, A, and trace elements. Genetic factors also play a vital role in the development of myocardial damage. Severe CFTR genotypes such as *ΔF508*: *ΔF508* and the asparagine-to-lysine mutation at position 1303 (N1303K) may be associated with the development of myocardial fibrosis and necrosis. Thus, myocardial fibrosis may result from the deficiency of trophic factors essential for myocardial metabolism against the background of genetic susceptibility to myocardial damage (severe CFTR genotypes together with the presence of certain modifying genes) [30,31]. Myocardial fibrosis leads to systolic [29] and diastolic [32] dysfunction, in both children [33] and adults [34,35], the gravity of which seems to correlate with the degree of hypoxemia and hypercapnia [26].

### 2.3. Coronary Artery Disease

Apart from the classically recognized right ventricular dysfunction, as life expectancy increases in CF patients, so does the risk of coronary artery disease, even though some experts believe that these individuals are “protected” from coronary atherosclerosis [36]. In fact, in addition to adult age, CF patients have several cardiovascular risk factors, such as chronic systemic inflammation, high fat diet, diabetes mellitus, and impaired lipid metabolism [36]. Furthermore, chronic inflammation found in CF can promote endothelial dysfunction and injury, increase arterial stiffness, and impair flow-mediated dilatation. Poore et al. examined brachial artery flow-mediated dilation in non-diabetic CF patients and found it to be reduced, a hallmark of endothelial dysfunction [37].

### 2.4. Endothelial Dysfunction

The *CFTR* gene is present in numerous locations throughout the cardiovascular system. Its presence in the coronary endothelium may lead to accelerated atherosclerosis, and pulmonary hypertension may be partially explained by impaired endothelial function in the pulmonary vasculature endothelium, where the CFTR gene is also present and may also be accountable for vascular remodeling and angiogenesis [18,38]. CFTR mutation present in the endothelium of peripheral vasculature may account for impaired vascular dilatation and increased stiffness [37]. Finally, CFTR is also present in cardiac myocytes, with a possible involvement in right and left ventricular dysfunction [39].

Arterial stiffness was found to be increased in children with CF who had reduced lung function by Eising et al. [40]. The authors investigated the aortic pulse wave velocity and found it to be increased, despite normal values of the blood pressure [8]. This results in increased afterload, which may worsen left ventricular dysfunction. CFTR inhibition in the pulmonary vascular bed is believed to escalate inflammation and neovascularization and vascular remodeling [41]. Furthermore, Wells et al. demonstrated that during pulmonary exacerbations, pulmonary artery diameter and pulmonary artery/aorta diameter ratio increased, a possible sign of pulmonary hypertension aggravation [42]. Bronchial artery endothelial dysfunction is presumed to increase the risk of hemoptysis [43].

Peripheral vasculature stiffness in CF was investigated by Hull et al. and Buehler et al. in adults and children, respectively. All age categories were demonstrated to have increased vascular stiffness, either by measuring pulse wave augmentation index or pulse wave velocity between the carotid and femoral arteries [44,45]. On the other hand, Skolnik et al. were unable to document any evidence of atherosclerosis or luminal narrowing in 14 adult CF patients suffering from diabetes or dyslipidemia. In contrast, others [18] have studied other chronic inflammation-associated conditions, such as idiopathic pulmonary fibrosis and rheumatoid arthritis, and found significantly more atherosclerotic burden compared to controls [35,46,47].

One possible explanation as to why coronary artery disease is so seldom described in CF patients is, however, the fact that these patients usually have low LDL levels due to lipid malabsorption. However, isolated cases of coronary artery disease or acute myocardial infarction have been previously described, especially in individuals over 45 [48,49]. Sandouk et al. published a mini case series of six patients with CFRD that developed coronary artery disease (non-ST segment elevation myocardial ischemia): two patients were smokers, four were hypertensive, and five patients were not receiving aspirin or statins prior to this event [50].

## 3. Cardiovascular Risk Factors in Cystic Fibrosis

**Figure 1:**
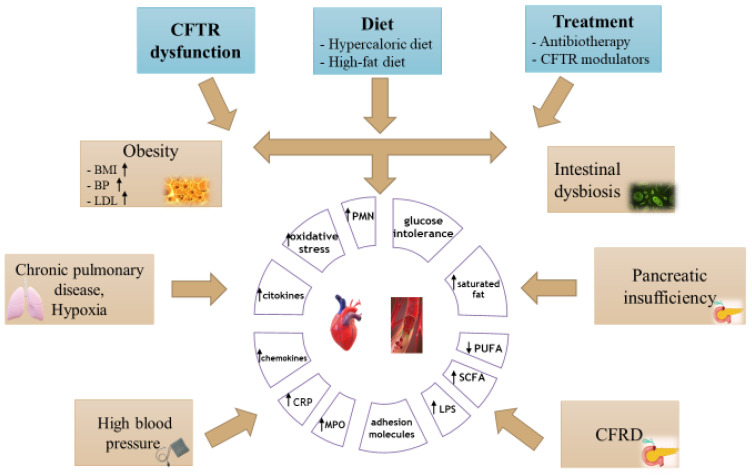
The pathogenesis of cardiovascular disease in cystic fibrosis (BMI—body mass index, BP—blood pressure, LDLc—low-density lipoprotein cholesterol, CRP—C-reactive protein, MPO—myeloperoxidase, LPS—lipopolysaccharides, SCFA—short-chain fatty acids, PUFA—polyunsaturated fatty acids, PMN—polymorphonuclear cells).

Patients with CF will experience several cardiovascular risk factors, among which we mention diabetes mellitus, high-fat diet, altered lipid metabolism, a decrease in the level of fat-soluble antioxidants, increased systemic inflammation, and therapeutic interventions. Chronic inflammation, along with increased levels of cholesterol and triglycerides, due to a diet rich in fat, will lead to the development of atherosclerotic disease. There are several possible modifiable risk factors in CF; however, the key contributing factors and critical age range for maximum impact of CF are still unknown. The introduction of novel CFTR modulator drugs might allow for more established cardiovascular risk factors to be targetable and preventable (Figure 1), (Table 2) [8,18,19,51].

## 4. Inflammation and Atherosclerotic Process in Cystic Fibrosis

Older adults have a higher rate of obesity, high blood pressure, and CFRD compared to young patients with CF due to their specific diet and drug therapies. In patients with CF, atherosclerosis may be accentuated by increased chronic inflammation and risk factor, specific to the disease. CF has both chronic systemic inflammation and organ inflammation. Proinflammatory states along with exaggerated responses to infections present a cumulative adverse effect at the cardiovascular level. Changes include several stages: damage to the vascular endothelium, deposition of low-density lipoproteins, proliferation of smooth muscle cells, and the development of atherosclerotic plaques. It has been observed that endothelial function parameters (level of cell-adhesion molecules, dilation, post-occlusive reactive hyperemia) have abnormal changes in pwCF [37]. At the arterial level, an abnormal function and structure was noted, with significant rigidity at the level of the aortic root and increased thickness at the level of the intima and the average at the carotid level, these aspects present especially in patients with pancreatic insufficiency [1,37].

A study that analyzed autopsy samples in patients aged between 6 and 13 years with and without CF noted the absence of atherosclerotic processes in these patients, due to the low concentration of circulating lipids that did not allow for the accumulation of intra-aortic lipids [52]. Another study looked at autopsy samples in older children between 10 and 24 years old with and without CF. In this category, the scale of the early atherosclerotic process was low, children with CF having fewer atherosclerotic lesions compared to healthy ones [19,53,54]. Therefore, we can conclude that aging contributes to the appearance of cardiovascular manifestations, along with other factors, which once accumulated cause pathological changes.

CF is characterized by systemic and organ-level inflammatory processes, determined by massive infiltration of PMNs (polymorphonuclears). These cells produce in addition to a wide spectrum of proinflammatory cytokines and also increased amounts of reactive oxygen and nitrogen species that appear to be involved in the progression of the disease. With the evolution of the treatment of CF, new targets have appeared, which involve neutrophil proteases, oxidizing agents, and the cascade of the inflammatory system, factors that cause the appearance of long-term complications. The atherosclerotic process is undoubtedly linked to inflammation and oxidative processes involving arterial walls. PMNs and different classes of monocytes have well-defined roles in the inflammatory process of atherogenesis, but besides these, other processes are involved whose roles remain unclear [9,19].

Reduced pulmonary function, along with inflammation localized in the respiratory tract, contributes to the appearance of cardiovascular changes. These changes, together with the inhalation of polluting particles containing redox-active species (metals, polyphenols, quinones) contribute to the amplification of the systemic inflammatory process, endothelial dysfunction, and disturbances of coagulation factors, leading finally to cardiovascular changes. The inflammatory process at the pulmonary level contributes to the triggering of several inflammatory biomarkers and thus to the maintenance of the systemic inflammatory process. Systemic inflammation is involved throughout the stages of the atherosclerotic process. Other systemic pro-inflammatory factors in patients with CF are induced by a diet high in saturated fat and insulin resistance with hyperglycemia. The systemic inflammatory response is revealed by the growth of circulating cytokines and chemokines and by the appearance of soluble adhesion molecules and acute phase reactants (C-reactive protein-CRP, fibrinogen), all of which contribute to a marked and persistent inflammatory process with proatherogenic risk [19].

The role of cytokines is to activate the function of circulating PMNs. Once activated, the circulating PMNs will release extracellular granular myeloperoxidase (MPO). In CF, it is known that there is an increased extracellular release of MPO when PMNs are activated [20]. Studies have shown the associations between increased levels of MPO and endothelial dysfunction, because the oxidative interaction of MPO with circulating lipid components and vascular wall tissues seems to have a role in triggering the atherosclerotic process [19,20].

Activating the inflammation will lead to increased amounts of oxidants involved in antimicrobial, adaptive, reparative processes, but in excess, they are known to cause damage to the already inflamed tissue. Oxidative stress is a balance disorder between the production of pro-oxidant agents and the antioxidant function of the host, having a key role in both CF and cardiovascular pathology. There are several factors that determine the appearance of oxidative stress in adults with CF such as: accelerated metabolism by producing large amounts of reactive species of mitochondrial oxygen, inflammatory processes, malabsorption of lipophilic antioxidants micronutrients and PUFA, as well as an inadequate metabolism of lipids and carbohydrates. In CF, oxidative stress is amplified by the appearance of diabetes or by exacerbations of the disease. The gold standard of oxidative stress and lipid peroxidation is the evaluation of isoprostans (products of arachidonic acid, omega-6 derivative). Several studies have observed an increase in 8-isoprostaglandin F2-alpha in the plasma and urine in patients with CF, which suggests a massive peroxidation of lipids and an imbalance between prooxidants and antioxidants in tissues and fluids. It is assumed that taking supplements with antioxidants would correct oxidative stress to some extent, but there is not enough evidence of clinical efficacy. These supplements seem to decrease oxidative stress biomarkers and increase quality of life, but their role in reversing lung function decline is still uncertain [1,19,20].

Diabetes in CF influences endothelial function, because it causes abnormalities in the tissues of the vascular wall, such as altered nitric oxide synthase function. Aging is a factor that causes the alteration of endothelial function as well as cardiomyocyte function by decreasing the available amount of nitric oxide [19,55]. It is necessary to understand the mechanisms of the appearance of endothelial dysfunction and subsequent CVD in pwCF.

## 5. Obesity Risk in Patients with CF

In recent decades, it has been shown that the weight status of patients with CF has improved considerably, but suitable nutrition remains a challenge. Some authors have expressed concerns about the increase in the prevalence of obesity, since an increased weight does not necessarily correlate with an improvement in the associated symptomatology. Overweight or obesity will lead to an impaired function of the respiratory muscles, decreased exercise tolerance, and immunological impairment. High-fat diets can exacerbate existing changes in intestinal microbial composition and chronic intestinal inflammation, with important clinical implications for people with obesity [5,56,57,58].

Regarding proteins and fats, people with CF require a higher intake (20% protein, 35–40% fat, 40–45% carbohydrates), which may lead to the development of long-term side effects.

It is known that obesity is frequently associated with multiple comorbidities that increase the risk of cardiovascular diseases: glucose intolerance, type 2 diabetes, dyslipidemia, hypertension, nonalcoholic fatty liver disease, increased inflammation, and metabolic syndrome. However, not all present with metabolic complications. They belong to metabolically healthy obesity (MHO) without any evidence of existing cardiometabolic disease. At the same time, there is another category of patients with metabolically unhealthy obesity (MUO) and an excess risk of adverse cardiometabolic outcomes and CVD [59]. Regarding obesity and CF, this brings up the question of whether pwCF present MHO or MUO. Weight gain and excess saturated fat contribute to the development of CVD, and excess adipose tissue in contrast to lean muscle mass will lead to the degradation of lung function and a negative prognosis of CF. Total cholesterol and LDL cholesterol levels tend to be low in CF, although elevations in triglycerides have been reported [5,36]. Harindhanuvadi et al. evaluated 484 adults with CF and found that the prevalence of hypertension was higher in overweight (25%) and obese (31%) pwCF, but total cholesterol levels and LDL levels were within the normal range [60]. In 2020, Bonhore et al. demonstrated an association between over-nutrition and higher blood pressure and LDL cholesterol, as well as insulin resistance in adult pwCF [61]. Elevations in lipid levels may be significantly common in pwCF with pancreatic sufficiency and after transplantation. The clinical significance of lipid abnormalities is currently unknown, but coronary artery disease may emerge as a complication of CFRD given the aging CF population [5,36]. As people with CF become more physiologically similar to the general population with the advent of CFTR modulator therapies, the impact of overweight and obesity on CF outcomes such as on pulmonary function, body composition, and their consequences for cardio-metabolic health warrants further investigation [62]. Currently, there is no recommended limit on cholesterol intake. Fats with unsaturated fatty acids should be used to avoid potential risk. In this regard, further studies are needed to establish the optimal protein and fat requirement in pwCF. Engelen et al. argue that determining the optimal protein and fat requirement depend on the condition of each individual, as well as on their energy consumption [63,64].

PwCF and pancreatic insufficiency (PI) are at risk of developing dyslipidemia. PI and disruption of fat absorption will lead to a deficiency of fat-soluble vitamins. Consequently, it will be necessary to take vitamin supplements together with foods rich in fat and pancreatic enzyme supplements to improve absorption, which will also contribute to the weight gain of patients [55,56].

Obesity in patients with CF will increase the risk of hypertension and the development of obstructive sleep apnea. It should also be noted whether obesity adversely affects the results of patients with CF undergoing lung transplantation. It has been observed that patients with an increased risk of malnutrition (those with severe mutations and PI) are at risk of becoming overweight or obese and developing CVD. As the survival rate of patients with CF increases under the treatment with appropriate modulators, the population undergoes an aging process that can also contribute to the appearance of cardiovascular manifestations even in the absence of obesity. Hypertension and increased cholesterol are especially worrying in this aging process, since these patients may experience additional cardiovascular factors (diabetes, inflammation). Although to date no deaths of cardiovascular cause have been reported in patients with CF, this aspect is of great interest and has resulted in increased vigilance towards screening and treatment. Further studies are needed to establish optimal targets for body mass index (BMI) values, proper nutrition, and maintenance of lung function [9,18,65,66,67].

The evaluation of nutritional objectives and the patients’ condition is recommended at each visit to adjust the recommended caloric intake and the exercise program. Recommendations should be made for each patient separately, considering their particularities, and they should be periodically reassessed. In addition, longitudinal studies are needed to describe the natural history of cardiovascular diseases in the adult population with CF, the effects of modulators on the cardiovascular system, as well as the effects of weight loss drugs and their effects on body composition [5,68].

## 6. Novel CFTR Modulator Therapies

Another important aspect is the treatment of CF with CFTR-modulating drugs. These drugs bring several benefits; however, all the effects they have on the body must be evaluated in the long term. Their role in improving lung function and increasing BMI has been demonstrated, but the increased weight of these patients can lead to unwanted long-term effects. Weight gain involves a series of changes in the cardiovascular system that are still being studied today to prevent potential conditions that may occur after treatment with CFTR modulators [57]. Combinations of CFTR modulators, Lumacaftor and Ivacaftor, were the first CFTR modulator drugs approved for the treatment of patients with CF homozygous for *F508del*. Even though the focus in recent decades has been on increasing BMI values to optimize lung function, the risk of obesity and overweight in the population with CF should be monitored in the long term and prevented. The mechanisms of modulators that underlie weight gain include decreased intestinal inflammation, decreased malabsorption, improved appetite, sense of smell and taste, and reduced energy consumption by relieving respiratory activity [51,57,65]. Furthermore, it was found that people with CF had increased levels of interleukin-6 (IL-6) and tumor necrosis factor alpha (TNF-a), which were involved in the development of atherosclerotic plaque and heart failure. Therefore, improved fat absorption resulting from the action of the CFTR modulator can contribute to the appearance of atherosclerosis and possibly increase insulin resistance, which will favor the appearance of metabolic syndrome [18]. Currently, the use of CFTR modulators is already known to bring benefits to the respiratory system and quality of life, and they require careful monitoring of the effect on the cardiovascular system [1].

## 7. Impact of Altered Gut Microbiota in Cystic Fibrosis

The gut microbiome, to a lesser extent, is inherited, and to a greater extent, it is influenced by environmental factors [66,69,70,71,72].

In pwCF, dysbiosis is promoted by antibiotic use, high-fat diet, and by the CFTR dysfunction, which leads to low intestinal pH through inadequate bicarbonate buffering, viscous, dehydrated secretions, decreased level of pancreatic enzymes, and delayed intestinal transit. Altered microbiota and local inflammation influence a child’s growth and quality of life and have an important role in gastrointestinal complications, such as malignancy. Data suggest that dysbiosis induces systemic inflammation and immune disorders that can lead to pulmonary exacerbations through the gut–lung axis [66,69,71,73].

The gastrointestinal tract is affected in most pwCF since intrauterine life and continues throughout childhood and into adulthood. Studies showed that the intestinal microbiota of pwCF differs from that of their healthy peers. Furthermore, researchers concluded that the intestinal microbiome of the patients affected by CF diversifies and matures with more difficulty compared to the healthy children [66,74]. Nielsen et al. concluded in their study that a CF child, even at fifteen years old, does not achieve the same microbiome richness as a healthy one-year-old child [75].

Ooi et al. observed that Ivacaftor positively influences the microbiome. This observation is strong proof that CFTR dysfunction influences the gut microbiota [76]. Furthermore, CF genotypes seemed to be associated with the severity of gut dysbiosis. For example, Schippa et al. demonstrated that the homozygous *F508* delta genotype is correlated with a more severe imbalance of the gut microbiota compared with other genotypes. Patients with this genotype had an abundance of *Escherichia coli* and a depletion of *Faecalibacterium prausnitzii* and *Bifidobacterium* [77].

An important alteration of the microbiome consists of decreased species diversity, modification associated with inflammatory processes (inflammatory bowel disease), metabolic disorders (obesity, type 1 and type 2 diabetes mellitus), or immune diseases (asthma) [66,78,79,80]. Data show that antibiotic use in pwCF is highly associated with impaired alpha diversity of the intestinal microbiome, both in the long and short term. In addition, there is evidence that antibiotics decrease Bifidobacterium concentrations throughout the intestine [81]. Although dysbiosis can be caused by a short antibiotic treatment, it is known to become severe with repeated antibiotic treatment. Burke et al. showed that the number of antibiotic therapies is correlated with the intestinal proportion of *Bacteroides*, which was decreased, and *Firmicutes* and *Veillonellacear*, which were increased [82,83]. Bruzzese et al. related that patients with CF included in their study who were on antibiotic treatment had significantly unbalanced microbiota, with a decreased proportion of *Bacteroides* and *Eubacterium*, compared with patients who did not follow an antibiotic therapy in the last 2 weeks [84]. In addition, Duytschaever et al. related that patients with CF are susceptible to a higher proportion of *Enterobacteriaceae,* which are resistant to Amoxicillin, as a result of the numerous Amoxicillin therapies that they have followed [85]. Manor et al. showed a higher prevalence of *Enterococcus faecalis* and *Enterococcus faecium*, species recognized to be susceptible to antibiotic resistance [86].

Another iatrogenic factor that could influence intestinal microbiota consists of proton pump inhibitor therapy. Proton pump inhibitor exposure is correlated with decreased diversity and an abundance of microorganisms that physiologically colonize the upper gastrointestinal tract. In addition, data showed an association between proton pump inhibitor therapy and a higher concentration of *Escherichia coli*, *Enterococcus* spp., and *Streptococcus* spp. [87,88].

Diet is another key factor that may alter the intestinal microbiome. CF patients need to follow a high-fat diet, a fact that leads to an increased abundance of *Firmicutes*, *Allobaculum*, and *Escherichia coli* and a decreased prevalence of *Akkermansia* and *Bacteroides* [89,90,91].

**Figure 2:**
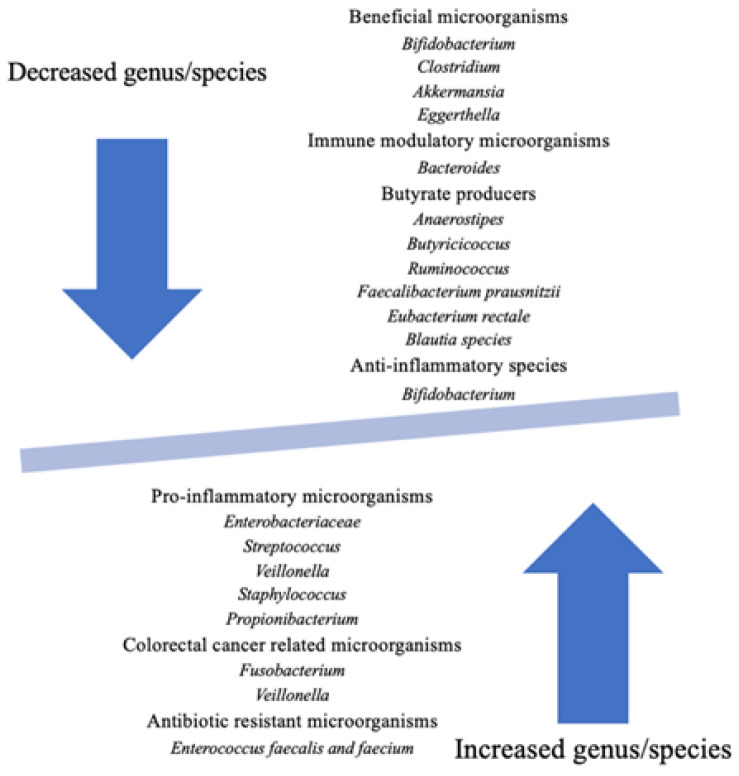
Gut dysbiosis in cystic fibrosis [74,79].

Numerous studies show decreased concentrations of *Bacteroides*, *Ruminococcaceae*, *Bifidobacterium*, *Roseburia*, and *Faecalibacterium* and increased concentrations of *Enterococcus*, *Veillonella*, *Firmicutes*, and *Proteobacteria* (*Escherichia*, *Shigella*, *Enterobacter*, and *Morganella*) in the microbiota of pwCF (Figure 2) [79].

Chronic intestinal inflammation in CF is objectified through fecal inflammatory markers and has a multifactorial etiology. Dysbiosis contributes significantly to promoting inflammation, as fecal studies showed reduced concentrations of microorganisms that have anti-inflammatory proprieties, such as *Faecalibacterium prausnitzii* and *Ruminococcaceae* family [66,79,92]. Decreased concentrations of bacteria that produce short-chain fatty acids (SCFAs) consist of another mechanism responsible for chronic inflammation apart from the well-known roles of SCFAs, maintaining the epithelial barrier, immunomodulation, and nourishment for intestinal cells [66].

There is evidence that alteration of the intestinal microbiome and inflammation are correlated with growth disorders. Hayden et al. showed that children with smaller height had a more imbalanced microbiome, characterized by a delayed maturation, compared to those with normal height of the same age. In addition, these patients had a reduced concentration of *Bacteroides* and an increased abundance of *Proteobacteria* [14]. Loman et al. concluded that a high prevalence of *Staphylococcus* and *Faecalibacterium* species is associated with low weight-for-length among children [93]. PwCF with CF are at risk of misusage of nutrients, as there is a lack of intestinal proteins that are essential for the transport and the metabolism of carbohydrates [66]. Literature data suggest that some bacterial species can influence the glucose homeostasis, such as *Alistipes* spp., which has a proven role in succinate metabolism [79].

## 8. Potential Role of Nutrition in Prevention of Cardiovascular Damage in Cystic Fibrosis

In addition to pharmacological therapy, CF management should also consider nutritional therapy. The current guidelines recommend that the goal of the diet is to develop a normal body weight and height similar to children without CF for those up to 2 years of age and for older children and adolescents to stay on the weight-, length- and BMI-for-age percentiles; the daily energy requirement of these patients has to be between 120% and 150%. As such, as many foods with increased energy intake as possible should be introduced into their diet, such as healthy fats from vegetables and seed nuts or additional proteins [63]. However, if we want to further improve the quality of life and the life expectancy in pwCF, early detection and intervention in preclinical CVD are necessary. It is critically important to revisit the classical hyper-caloric, high-fat CF diet, because the new CFTR modulators offer substantial benefits to respiratory health and quality of life, but their effects on CVD risk are largely unknown. In order to maintain a nutritional status as close as possible to the physiological one, patients with CF must pay special attention to food and eating habits and benefit from appropriate nutritional counseling, which is essential in the evolution and prognosis of the disease [94]. The qualitative improvement of the diet of pwCF with nutrient-dense foods, following the recommendations of the current guidelines, with an appropriate intake of fruits and vegetables, vegetable proteins, and sea-foods, will result in a better intake of bioactive food components, which brings the potential benefits of nutraceuticals to pwCF.

The introduction of CFTR modulator treatment currently requires the modification of classic hyper-caloric, high-fat diets with higher quality diets. Patients using CFTR modulator therapies have a favorable evolution of CF but present an increased risk for obesity. In a study published in 2018, Sutherland et al. showed that energy-dense, nutrient-poor foods were widely consumed by those receiving a high-energy, high-fat CF diet, an aspect also correlated with socio-economic and demographic factors, as well as school attendance, whereby the parents had no control over feeding. The study emphasized that the CF diet must be respected and personalized, and it is necessary to find a balance between nutrient-dense and energy-dense, nutrient-poor foods sources to obtain the optimal nutritional status [95].

A multicenter, cross-sectional, observational European study published in 2019 by Calvo-Lerma et al. evaluated the relative contribution of food groups in terms of nutrient intake in children with CF. The study results indicated inadequate nutritional profiles, with low amounts of PUFA and MUFA and high intake of SFA and sugars. This nutritional imbalance was due to the high consumption of meat, dairy, and processed products and low consumption of fish, nuts, and legumes [96].

Starting from the recommendations of the initial CF guidelines, McDonald et al. emphasize the importance of a balanced diet by age category, which has been shown to have a positive impact among non-CF patients; this diet should contain vegetables, fruits, whole grains, seafood, eggs, beans, peas, nuts and seeds, dairy products, and meat and poultry. Practically, these recommendations make the transition from the quantitative historical diet to the current, qualitative one, the goal being a personalized nutritional management [97,98].

Food contains many bioactive products, which have proven their benefits in various pathologies, such as the prevention and reduction of CVD risk [99], diabetes prevention and management [100], and the prevention of non-alcoholic fatty liver disease [101,102] and obesity and its relationship with other diseases [103]. Increasing evidence in the last years shows that many bioactive food components, such as phytochemicals, polyunsaturated fatty acids, and antioxidants have favorable effects in the management of CF. As a result, we are trying to use these bioactive components from both food and food supplements in the nutritional management of CF.

### 8.1. Phytotherapy in Cystic Fibrosis

**Figure 3:**
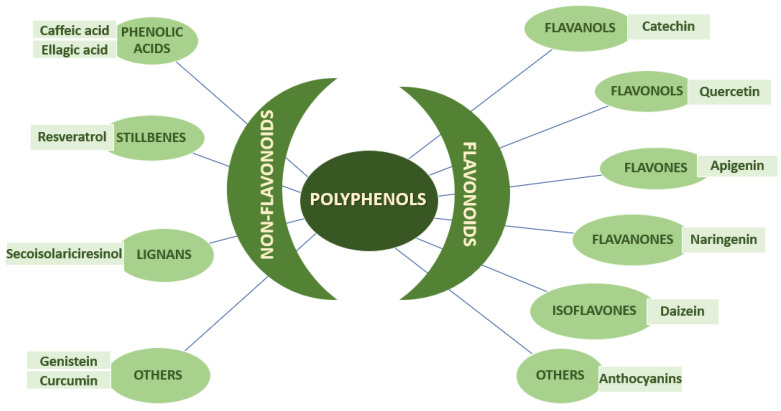
Classification of polyphenols (adapted by [105]).

Phytochemicals are chemical compounds that we find naturally in plants such as different fruits, vegetables, tea, coffee, cocoa, mushrooms, and traditional medicinal herbs. At present, according to the numerous studies published in the specialized literature, many bioactive food or plant components are considered potential therapies in reducing cardiovascular risk factors. Food polyphenols are classified into four categories depending on the number of phenolic rings and the structural elements to which the phenolic nucleus is attached: flavonoids, phenolic acids, stilbenes (resveratrol), or lignans (Figure 3) [104].

Nutraceuticals with potential benefits in reducing cardiovascular risk factors are presented in Table 3.

### 8.2. Quercetin

Quercetin is apparently one of the flavonoids abundantly present in the human diet, in fruits and vegetables such as garlic, onions, green beans, broccoli, cabbage, tomatoes, berries, apples, and green tea. It is known to have anti-inflammatory, antioxidant, antiviral, anti-carcinogenic, and anti-platelet aggregation effects [118]. In 2010, the U.S.A. Food and Drug Administration (FDA) granted the status of “generally recognized as safe” (GRAS) to a highly pure form of quercetin [119].

In 2015, Peters et al. showed the impact that quercetin, resveratrol, and epicatechin have on CF; the conclusion to which they came was that CFTR plays an important role in the maintenance of vascular homeostasis, and that is how the vasoprotective role of polyphenols would be explained [120].

At the intestinal level, quercetin was identified as a modulator of calcium-activated chloride channels’ (CaCCs) activity, and currently, it is known that CFTR and CaCCS are the main chloride channels in the luminal membrane of enterocytes. Knowing this, quercetin could represent a potential therapy in CaCC-related diseases [121].

The administration of other polyphenols such as apigenin, kaempferol, and genistein proved to have positive effects on the intracellular distribution of CFTR with 24 h treatments [106]. In human clinical trials, flavonoids have proven to be well tolerated, which is why they are considered to be possible therapeutic agents in CF [122].

In a study published in 2017 in which the authors studied the effects of resveratrol, curcumin, and quercetin on cell culture models of obesity, animal models of obesity, human studies, and clinical trials, these substances proved their beneficial effects in obesity therapy by reducing intracellular oxidative stress, decreasing chronic inflammation, and inhibiting the adipogenesis and lipogenesis processes. However, in order to introduce quercetin in human treatments, more extensive studies are needed to accurately determine the high-dose and long-term usage [123].

Quercetin has been clinically proven to induce a significant decrease in systolic blood pressure, but it had no effect on other cardiovascular risk factors and inflammatory biomarkers [107]. Among the known properties of polyphenols or polyphenol-rich diets, there are also cardiovascular, antioxidative, and anti-inflammation effects by decreasing the production of reactive oxygen species, decreasing the production of superoxide, blocking the formation of oxidized low-density lipoprotein [124], and playing a potential role in cytokine expression and endothelial inflammatory markers as well as increased NO production [108].

In an article published in 2019, Ditano-Vázquez et al. showed the cardiometabolic benefits offered by a diet with low-to-moderate consumption of red wine with meals and virgin olive oil, which contains a high concentration of polyphenols; the study strongly supports the positive influence of the Mediterranean diet over inflammation markers, oxidative stress, improvement of lipid serum level, insulin sensitivity, and endothelial function [125].

Discussing cardio-metabolic risk factors and the effects of polyphenol consumption, Giacco et al. shows that a daily nutritional intake of polyphenols could be recommended according to current nutritional guidelines for the general population and also for pwCF; however, additional studies are needed to certify the effectiveness of polyphenols in the management of cardio-metabolic risk factors, because the currently existing evidence does not allow for the recommendation of their use as supplements to reduce type 2 diabetes mellitus (T2DM) and CVD risk [126]. A regular food intake ensures a daily amount of 5–100 mg quercetin/day; the consumption of foods with a high content of quercetin will bring an intake of up to 500 mg/day. Absorption is increased during meals richer in lipids or in combination with apple pectin, oligosaccharides, and lecithin. Most clinical studies use a total daily dose of 500–1000 mg/day [127].

### 8.3. Curcumin

Turmeric, scientifically known as Curcuma longa, is an Asian spice whose major polyphenols are curcumin, demethoxycurcumin, and bisdemethoxycurcumin, the most active being curcumin. Its unique chemical structure gives it a strong antioxidant role, which is joined by other functions such as inhibiting the metabolism of arachidonic acid and the activity of TNF-alpha, IL-6, lipoxygenase, and cyclooxygenase [128]. In a literature review on the anti-obesity effects of different polyphenols, studies involving treatment with curcumin in a dose of 1 g/day were presented. In a group of obese patients, curcumin showed to have lowering effects on triglyceride levels as a sign of improved insulin sensitivity. In another studied group, after a 30-day treatment, lower levels of IL-1 beta and IL-4 were recorded among obese subjects, and in a randomized control trial involving patients with metabolic syndrome, treatment with curcumin led to a reduction in weight and abdominal fat tissue [129]. Another review focused on the use of curcumin in type 2 diabetes mellitus (T2DM) at safe doses up to 12 g per day in adults. The data from clinical trials showed that curcumin had lowering effects on C-peptide, endothelin-1, IL-6, TNF-alpha, LDL, VLDL, triglycerides, HbA1c, serum glucose, and leptin. At the same time, an increase in adiponectin, HDL, and lipoprotein lipase activity was observed [130]. Benefits of curcumin have also been found for the cardiovascular system, through inhibition of the JAK/STAT signaling pathway and the inhibition of atheroma plaque formation [131]. Another vascular effect of curcumin administration is to decrease pulse wave velocity [132]. Curcumin’s implications in CF are disputed; it is supposed that curcumin increases the activity in CFTR-regulated channels. In 2004, Egan et al. published a study on murine models with *deltaF508* mutation in the CFTR protein, one of the common CF-associated mutations, concluding that curcumin acting as a SERCA pump inhibitor (sarcoplasmic/endoplasmic reticulum calcium pump inhibitor) could increase the number of functional *deltaF508 CFTR,* which makes curcumin potentially useful in CF treatment [133]. There is also a class III mutation, the glycine to aspartate missense mutation at the 551 position, where curcumin can act by increasing the G551D-CFTR opening channel. Studies have shown that due to the reduced bioavailability of curcumin, when combined with another polyphenol such as genistein, it has a greater action on G551D-CFTR gating defects [134,135].

Genistein is a heterocyclic polyphenol found mainly in soy that acts as a phytoestrogen, tyrosine kinase inhibitor, and antioxidant. Its role in CF is as a potentiator for curcumin, augmenting the activity of G551D-CFTR channels [136]. In addition to the roles mentioned, there are studies that support the antibacterial activity of curcumin through upregulation of toll-like receptor-2 (TLR2). TLR2, a receptor found in the epithelial cells for the peptidoglicans from pathogenic bacteria, has a reduced activity in pwCF, thus contributing to risk of infection [109].

### 8.4. Resveratrol

Resveratrol is known as a pluripotent non-flavonoid polyphenol—an antidiabetic compound and anti-inflammatory, antioxidant, antiapoptotic, antifibrotic, antihypertensive, and antitumoral agent, with effects on the liver and kidneys and a role in neurodegenerative diseases [137]. Rich sources of resveratrol are dark-colored fruits such as grapes, blackberries, plums, blueberries, and cranberries, but also peanuts, pistachios, and soy. Another rich source is red wine and Itadori tea [138]. A systematic review and meta-analysis in 2022 demonstrated the role of resveratrol in maintaining blood glucose in normal values as well as in improving cardiometabolic parameters in T2DM [110].

The effects of resveratrol in obesity were highlighted in ex vivo studies, in which after acting on the expression of Peroxisome Proliferator-activated Receptor y (PPARy) and sirtuin 1, the result was the inhibition of adipogenesis and the prevention of triglycerides increase; limited data from clinical trials show a decrease in inflammatory status in obese patients and an improvement in metabolic status in those with T2DM [111].

Regarding CF, a well-known aspect is that patients have a higher level of oxidative stress compared to the healthy population. These patients present an infection–inflammation cycle that results in increased proinflammatory status, generated by oxidative stress, for which the deficit of CFTR is responsible [139]. Understanding how resveratrol is beneficially involved in CF pathogenesis could represent one possibility for treatment and lead to a better understanding of CF cell signaling [112].

### 8.5. Allicin

Allicin (diallylthiosulfinate) is a bioactive compound from garlic (*Allium sativum* L.) with a wide range of biological effects. In a review article published in 2022 that included 26 studies, the positive effect on the CF evolution of nutraceuticals, including micronutrients (vitamins, magnesium, zinc), fibers, proteins, and aminoacids, lipids, phytochemicals (apigenin, genistein, quercetin, curcumin, allicin), were discussed. The emergence of nutraceuticals in the food industry and the studies that highlight the impact and benefits they bring to patients could be a new approach in the management of CF [113]. Allicin proved effective in improving metabolic disorders in obese mice that received a high-fat diet; a reduction in body weight was observed, increasing thermogenesis and lipolysis and insulin sensitivity. Further, the modification of the intestinal microbiota secondary to the administration of allicin was noted, in the sense of increasing the proportion of beneficial bacteria [114]. Another study showed that allicin helps to reduce body weight by reducing adiposity, increasing energy consumption, and activating brown adipose tissue, as well as maintaining glucose homeostasis [115]. In addition, through modulation of the intestinal microbiota, another article showed the benefits of raw garlic juice and allicin supplementation against cardiovascular risk factors by reducing the formation of trimethylamine-N-oxide originating from the metabolism of L-carnitine [116]. Another study showed the role of garlic extract in reducing blood pressure in hypertensive persons, improving lipid profile and immune status [117].

These observations make allicin or garlic extracts a possible therapeutic option to prevent and/or treat obesity and related metabolic disorders in the future. Data from specialized literature show that phytochemicals, mainly represented by curcumin, quercetin, resveratrol, and allicin represent a new potential therapeutic path, with multiple benefits for pwCF and in prevention of CVD.

Other nutraceuticals with beneficial effects in reducing cardiovascular risk are green tea, red yeast rice, cocoa, soy, and lycopene.

### 8.6. Green Tea

The second most consumed beverage in the world is tea. The known antioxidant and anti-inflammatory benefits of green tea are due to the abundance of catechins in its composition, which have an anti-atherosclerotic effect and reduce lipid peroxidation. The constant consumption of green tea has proven its effectiveness in reducing total cholesterol, LDL-cholesterol, and oxidized low density lipoproteins and had an important antihypertensive effect [140,141]. Other benefits of using green tea are the reduction of lipid absorption at the intestinal level and implicitly the improvement of lipid profile, the prevention of inflammation at the vascular level involved in the progression of atheromatous lesions, and the activation of endothelial nitric oxide involved in the regulation of vascular tone [142].

### 8.7. Red Yeast Rice

Red yeast rice is used in food in certain areas of Asia, as well as in traditional Chinese medicine; it is a natural product of yeast (Monascus purpureus), grown on white rice. The active substance in red rice yeast is “monacolin K”, with the effect of maintaining normal concentrations of cholesterol and triglycerides in the blood by inhibiting of 3-hydroxy-3-methylglutharyl-coenzyme A (HMG CoA) reductase [143]. Other benefits include reducing oxidative stress by decreasing the formation of free oxygen radicals, anti-inflammatory qualities, stabilizing atheroma plaque, and changing biological markers involved in inflammation and vascular remodeling [144].

### 8.8. Cocoa

Cocoa has a rich content of polyphenols of the flavanol type. Daily dietary interventions with foods rich in flavanols, such as dark chocolate, are correlated with an important reduction in systolic blood pressure and cardiovascular risk [145]. Further, cocoa consumption is correlated with improving the blood lipid profile, platelet activity, and endothelial function, as well as positive antioxidant and anti-inflammatory effects [146].

### 8.9. Polyunsaturated Fatty Acids

Polyunsaturated fatty acids (PUFA) are essential components that the body cannot synthesize on its own, but must take them from the diet. The family of long-chain polyunsaturated fatty acids includes omega-3 fatty acids, represented by alpha linoleic acid (ALA), decohexanoic acid (DHA), and eicosapentanoic acid (EPA). Their administration could play an extremely important role in preventing CVD in CF. Another class of polyunsaturated fatty acids is represented by the omega-6 family that includes linoleic acid (LA), gamma-linoleic acid (GLA), and arachidonic acid (AA) (Table 4) [147,148].

Long-chain fatty acids, especially DHA, are structural components of cell membrane phospholipids. In FC, there is a deficiency of essential fatty acids due to the presence of disorders of fatty acid metabolism and malabsorption of fats (despite the replacement with pancreatic enzymes). This will lead to an imbalance between omega-3 LC-PUFA and omega-6 LC-PUFA in the cell membrane, resulting in inhibition of the anti-inflammatory pathway mediated by omega-3 metabolites’ LC-PUFA and an exacerbation of the inflammatory pathway mediated by metabolites of arachnidonic acid (most commonly omega-6 LC-PUFA) (Table 4) [148,149,150,151,152].

### 8.10. Vitamins

It is now well known that all pwCF receive the fat-soluble vitamins A, D, E, and K from the moment of diagnosis in the doses recommended by current guidelines. However, with the change in the pattern of the disease and the increase in the risk of obesity and cardiovascular complications, the question arises as to whether other vitamins with cardioprotective roles should be administered. Some liposoluble vitamins such as A, D, and E, as well as hydro-soluble vitamins such as B6 (pyridoxine), B9 (folic acid), and C have been reported to play a major role in modulating the cardiovascular function [153,154,155,156]. Several experimental and observational studies have revealed the usefulness of different vitamins in CVD [157,158,159,160,161,162,163,164]. Links between different vitamins and CVD have been demonstrated based on their effects on changes in the levels of inflammation, oxidative stress, homocysteine, lipoproteins, and nitric oxide. Studies on vitamins and cardiovascular or metabolic diseases have shown that although they do not have a direct effect on the cardiovascular system, for example, the fat-soluble vitamins influence the risk factors for the cardiovascular pathology, which underlines the idea that the favorable effects on CVD are due to the correction of deficiencies [153].

Vitamin A is highly concentrated in multiple cereals, orange and yellow vegetables, many fruits, and in olive and fish oils. Supplementing the diet with vitamin A has shown to have lowering effects on blood pressure for hypertensive patients [165] and preventive effects against hypertension at higher intake doses [166]. Studies on animal and human subjects have shown the improvement of atherosclerosis due to the antioxidant and anti-inflammatory role of this vitamin, a significant reason for consuming natural oxidants, e.g., B-carotene, which plays a role in reducing CVD [167,168].

Vitamin D has beneficial implications for skeletal development as well for the prevention of cardiovascular risk factors [169]. Though suppression of the parathyroid hormone can lead to enhanced lipolysis and increasing calcium levels can lead to a reduction in triglyceride formation in the liver, all these effects contributing to a decrease in serum cholesterol levels and triglyceride levels reduce the occurrence of dyslipidemia [170]. Vitamin D can also contribute to obesity reduction by two mechanisms: uncoupling the oxidative phosphorylation in adipose cells, thus leading to increased energy consumption, and promoting the mobilization of free fatty acids from fat cells [171]. On the other hand, there are studies that have shown that in patients with metabolic syndrome, vitamin D did not lower serum lipids and HBA1c and only had lowering effects on blood pressure and insulin resistance [172]. Due to its renin-angiotensin system inhibition, it can contribute to hypertension control; its contribution to this cardiovascular pathology is also sustained by its role in vasoconstriction and vascular calcification reduction [173].

Vitamin E, and particularly α-tocopherol, is found in plant-based oils, seeds, and many fruits and vegetables. Vitamin E acts as a potent antioxidant, being able to interfere in the pathogenesis of several diseases, such as cardiovascular events, obesity, and diabetes through its ability to protect the endothelial barrier functions, regulate lipid homeostasis, modulate serum lipids level, and prevent low-density lipoprotein oxidation [174].

Studies on vitamin K effects in cardiovascular events have shown that a diet rich in foods containing this micronutrient may reduce atherosclerotic cardiovascular disease [175]. Its implication in the cardiac and vascular pathology is due to role played in the matrix Gla protein activation, an extrahepatic γ-carboxyglutamate protein that was found to be the most potent vascular calcification inhibitor [176].

The vitamin B group has also been studied for its beneficial effects on the cardiovascular system. As the fat-soluble vitamins mentioned, vitamin B has also been recognized as a molecule that is able to lower blood pressure, and in combination with folic acid, it had the ability to decrease the damage of the myocardial cells produced by isoproterenol. In animal studies, these micronutrients recorded promising results in lowering oxidative stress levels [153,177].

Ascorbic acid is an essential water-soluble vitamin that cannot be synthesized by the organism, so it is obtained exclusively through diet. The sources of vitamin C are fresh fruits and vegetables, and the principle ones are citrus fruits, berries, tomatoes, and green leafy vegetables [178]. The main benefits of vitamin C consist of it having anti-inflammatory properties and being an important dietary antioxidant by reducing reactive oxygen species and free radicals, and furthermore, it promotes the activity of other antioxidants, such as glutathione and α–tocopherol (vitamin E) [179]. Plasmatic concentrations of ascorbic acid are inversely associated with body mass index, body fat, and waist circumference. Moreover, vitamin C intake is associated with lower blood glucose, triglycerides, LDL-cholesterols, and insulin resistance, but also with an increased fat oxidation, suggesting a relationship between ascorbic acid level and adiposity [180]. Several studies have concluded that decreased levels of vitamin C are associated with high blood pressure, endothelial dysfunction, and atherosclerosis [179]. Furthermore, many studies have shown that vitamin C promotes and protects endothelial functions. The role of vitamin C in nitric oxide synthesis has been demonstrated in cultured human endothelial cells. This property can protect the vessels from vasoconstriction, atherosclerosis, and coagulation dysfunctions [181,182].

## 9. Conclusions

In addition to the pharmacological approach, dietary intake plays an essential role in the evolution and prognosis of patients with cystic fibrosis, both in the underlying disease and in the complications that arise. The possibility of preventing/reducing cardiovascular risk in these patients contributes significantly to improving the quality of life and increasing life expectancy in the long term. Until recently, the most frequently encountered association with CF was malnutrition, but currently, there are also cases of CF with obesity, with all the associated risks.

Nutritional therapy is required to be adapted to these new pathological associations, starting from the recommended macro- and micronutrient requirements in cystic fibrosis, with the necessary adjustments to improve the identified cardiovascular risk factors. The opportunity to reduce cardiovascular risk through nutrition in a chronic disease with genetic determinism is still in a theoretical stage. Research in recent years has also focused on the actions of food and plant bioactive compounds, and the results showed that nutraceuticals represent a possible therapeutic path, with many studies pointing towards their anti-inflammatory and antioxidant properties and their role in reducing body mass index, normalizing the lipid profile, and improving endothelial function. Data from specialized literature shows that phytochemicals, mainly represented by curcumin, quercetin, resveratrol, and allicin, represent a new potential therapeutic path, with multiple benefits for cystic fibrosis patients and in the prevention of cardiovascular disease. Most studies were carried out on experimental animals; therefore, large clinical trials are necessary to accurately establish potential cardioprotective dietary products.

This study has one major limitation: the majority of the data described in the section highlighting the role of bioactive food substances in CVD do not refer to individuals with CF. This is due to the fact that few studies regarding the optimal nutrition of pwCF have so far been performed.

## Figures and Tables

**Table 1:** Current understanding of CFTR presence, clinical impact, and potential management options for primary and secondary cardiovascular complications in CF adapted by Poore, Taylor-Cousar, and Zemanick, 2022 [1,9,18,19,20] (CF: cystic fibrosis, RV: right ventricle; LV: left ventricle; CFRD: cystic fibrosis-related diabetes; IGT: impaired glucose tolerance; iNO: inhaled nitric oxide).

Anatomy	Clinical Impact	Management Options
Heart	RV and LV dysfunction	Exercise Glycemic control for CFRD/IGT In selected cases: anti-fibrotic agents such as spironolactone or angiotensin-converting enzyme inhibitors, positive inotropic agents
Coronary Arteries	Atherosclerosis	Exercise Glycemic control for CFRD/IGT Dietary modifications Oxygen iNO Sildenafil Sleep optimization Statins Coronary stenting
Pulmonary Vasculature	Pulmonary hypertension Angiogenesis/remodeling Enlarged pulmonary arteries Increased levels of pro-oxidant cytokines	Pulmonary hypertension-specific treatment Sildenafil Fasudil Lung transplantation
Peripheral Vasculature	Impaired dilatation Increased stiffness Impaired endothelial function Blood pressure differences ROS production Dysregulate NOS activities	Exercise CFRD treatment/screen Flavonoids Calcium channel blockers PUFA, vitamin E Blood pressure monitoring Salt intake and hydration

**Table 2:** Potential cardiovascular risk factors in people with cystic fibrosis [1,8,18,19,51].

Non-Modifiable Risk Factors
Chronic inflammation Pro-oxidative states CFTR-related vascular dysfunction Pulmonary hypertension Chronic pulmonary, cardiac, and renal disease Hyperglycemia and CFRD Pro-inflammatory macrophages Increased levels of cellular adhesion molecules CFTR-related plaque instability
**Modifiable Risk Factors**
High-calorie, high-fat diet Lung infection Solid-organ transplant Novel CFTR modulator therapies Indwelling vascular devices Pro-thrombotic coagulation

**Table 3:** Nutraceuticals with potential benefits in reducing cardiovascular risk factors [104,105,106,107,108,109,110,111,112,113,114,115,116,117]. (LDL—cholesterol-low density lipoprotein cholesterol, Hs CRP—high-sensitivity C-reaction protein, TNF-alpha—tumor necrosis factor-alpha, IL-6—interleukin-6, HDL-cholesterol—high-density lipoprotein cholesterol, HbA1c—glycated hemoglobin, HOMA index—Homeostatic Model Assessment of Insulin Resistance index.)

Nutraceuticals	Clinical and Biological Effects
**RESVERATROL**	Anti-inflammatory, antioxidant, antiapoptotic, antitumoral, antifibrotic, antidiabetic Effects on laboratory risk markers—not demonstrated Decreased blood pressure
**CURCUMIN**	Antioxidant, anti-inflammatory Lowering effects on triglyceride and LDL-cholesterol levels; improved insulin sensitivity; decreased leptin, Hs CRP, TNF-alpha, IL-6; increased HDL-cholesterol, adiponectin Decreased pulse wave velocity
**QUERCETIN**	Anti-inflammatory, antioxidant, antiviral, anti-carcinogenic, anti-platelet aggregation Inhibited the adipogenesis and lipogenesis processes, decreased serum level of triglyceride and total cholesterol Reduced systolic blood pressure
**ALLICIN/AGED GARLIC EXTRACT**	Reduced body weight, increased thermogenesis and lipolysis and insulin sensitivity Reduced total cholesterol and LDL-cholesterol Reduced blood pressure
**GREEN TEA**	Decreased blood lipids, reduced ischemia/perfusion injury Improved endothelial functions Anti-inflammatory and antioxidant
**RED YEAST RICE**	Reduced LDL-cholesterol, Apolipoprotein B, Hs CRP Improved endothelial functions Anti-inflammatory and antioxidant
**BEETROOT JUICE**	Reduced oxidate stress, inflammation Reduced blood pressure
**COCOA**	Anti-inflammatory and antioxidant Improved endothelial functions, platelet activity Improved blood lipids profile; reduced glucose, HbA1c, HOMA index Reduced systolic blood pressure

**Table 4:** Classification, sources, and roles of LC-PUFA [148,149,150,151,152].

Name	Sources	Roles
	Omega-3 LC-PUFA	
Alpha linoleic acid (ALA)	- Essential acid (it cannot be synthesized by the body, is taken from the diet) - Fatty fish, fish oil, nuts, soy, beans, beans, rapeseed, flaxseed, plants	- Reducing triglyceride levels (by decreasing the hepatic synthesis of VLDL particles and increasing the activity of protein lipase), decreasing the inflammatory process, decreasing platelet aggregation, and stabilizing the atheroma plaque; preventing diabetes, arrhythmias and coronary events; optimal neuronal and retinal function.
Decohexanoic acid (DHA)	- Fatty fish, fish oil, avocado	The same roles as ALA.
Eicosapentanoic acid (EPA)	- Fatty fish, fish oil, avocado	The same roles as ALA.
	Omega-6 LC-PUFA	
Linoleic acid (LA)	- Essential acid (it cannot be synthesized by the body, is taken from the diet) - Corn and sunflower oil, margarine, almond	- LA generates anti-inflammatory molecules; at the level of the vascular endothelium, it presents anti-inflammatory properties, suppressing the production of adhesion molecules, chemokine, and interleukins that are mediators of the atherosclerotic process. - The increased values of the omega-6/omega-3 ratio facilitate the development of cardiovascular diseases, inflammatory, autoimmune diseases, and even cancer.
Gamma-linoleic acid (GLA)	- Most foods	- Promoting inflammation and increasing cardiovascular risk. - The increased values of the omega-6/omega-3 ratio facilitate the development of cardiovascular diseases, inflammatory, autoimmune diseases, and even cancer.
Arachidonic acid (AA)	- Meat and eggs	- Promoting inflammation (is involved in the early stages of inflammation) and increasing cardiovascular risk. - The increased values of the omega-6/omega-3 ratio facilitate the development of cardiovascular diseases, inflammatory, autoimmune diseases, and even cancer.

## Data Availability

Not applicable.
